# Mental Health Status of Late-Middle-Aged Adults in China During the Coronavirus Disease 2019 Pandemic

**DOI:** 10.3389/fpubh.2021.643988

**Published:** 2021-05-26

**Authors:** Yong-Bo Zheng, Le Shi, Zheng-An Lu, Jian-Yu Que, Kai Yuan, Xiao-Lin Huang, Lin Liu, Yun-He Wang, Qing-Dong Lu, Zhong Wang, Wei Yan, Ying Han, Xin-Yu Sun, Yan-Ping Bao, Jie Shi, Lin Lu

**Affiliations:** ^1^Peking University Sixth Hospital, Peking University Institute of Mental Health, National Health Commission (NHC) Key Laboratory of Mental Health (Peking University), National Clinical Research Center for Mental Disorders (Peking University Sixth Hospital), Chinese Academy of Medical Sciences Research Unit, Peking University, Beijing, China; ^2^Peking-Tsinghua Center for Life Sciences and PKU-IDG/McGovern Institute for Brain Research, Beijing, China; ^3^Savaid Medical School, University of Chinese Academy of Sciences, Beijing, China; ^4^Peking University Health Science Center, National Institute on Drug Dependence and Beijing Key Laboratory of Drug Dependence, Peking University, Beijing, China

**Keywords:** COVID-19, late-middle-aged adults, mental health, prevalence, risk factors

## Abstract

**Background:** The novel coronavirus 2019 (COVID-19) pandemic and related compulsory measures have triggered a wide range of psychological issues. However, the effect of COVID-19 on mental health in late-middle-aged adults remains unclear.

**Methods:** This cross-sectional, web-based survey recruited 3,730 participants (≥ 50 years old) between February 28 and March 11 of 2020. The Patient Health Questionnaire-9, Generalized Anxiety Disorder-7, Insomnia Severity Index, and Acute Stress Disorder Scale were used to evaluate depression, anxiety, insomnia, and acute stress symptoms. Multivariate logistic regression analysis was fitted to explore risk factors that were associated with the selected outcomes.

**Results:** The mean age of the participants was 54.44 ± 5.99 years, and 2,026 (54.3%) of the participants were female. The prevalence of depression, anxiety, insomnia, and acute stress symptoms among late-middle-aged adults in China during the COVID-19 pandemic was 20.4, 27.1, 27.5, and 21.2%, respectively. Multivariable logistic regression analyses showed that participants who were quarantined had increased odds ratios for the four mental health symptoms, and those with a good understanding of the COVID-19 pandemic displayed a decreased risk for all mental health symptoms among late-middle-aged adults. In addition, participants with a low income and with a risk of COVID-19 exposure at work had a remarkably high risk of depression, anxiety, and acute stress symptoms.

**Conclusions:** Mental health symptoms in late-middle-aged adults in China during the COVID-19 pandemic are prevalent. Population-specific mental health interventions should be developed to improve mental health outcomes in late-middle-aged adults during this public health emergency.

## Introduction

The novel coronavirus 2019 (COVID-19) outbreak began in December of 2019 and became an international public health emergency. COVID-19 is highly contagious and spreads quickly (1). More than 73 million people were infected with COVID-19, and 1,663,474 patients died worldwide as of December 19, 2020 (2). To control the escalation of the pandemic, governments have implemented several restrictive measures, including screening programs, control and containment measures, and quarantine strategies (3–5). The devastating consequences of the COVID-19 pandemic and compulsory measures that place people in isolation may trigger a wide range of psychological issues (6, 7). The identification of people who are at risk for developing mental health symptoms during the COVID-19 pandemic is important for policy making and medical resource allocation.

Based on published data, COVID-19 affects late-middle-aged adults more frequently than children and young adults (8). The geriatric population is generally more susceptible to severe illness and has a high mortality rate, ranging from 15 to 20%, because of more prolonged recovery and a faster progression of comorbidity caused by COVID-19 (9–11). Due to their relatively lower utilization of online social media, late-middle-aged adults may be sensitive to isolation and loneliness that are consequences of restrictive measures such as traffic restrictions and quarantine (12–15). Thus, they may suffer from more psychological stress during the COVID-19 pandemic; however, restrictive measures limit their access to mental health assistance (16). Moreover, previous studies found a high prevalence of mood and anxiety disorders and a heavy mental disorder burden in late-middle-aged adults, making them a vulnerable population for mental illness during COVID-19 (17, 18). The World Health Organization has warned that the risks of COVID-19 may generate greater mental health symptoms in these individuals during the pandemic and should receive more attention (19).

During other epidemics involving respiratory pathogens, such as severe acute respiratory syndrome, psychological symptoms among the geriatric population raised great concerns, and several personal and epidemic-related factors were associated with mental illness in late-middle-aged adults (20, 21). A recent study analyzed the psychological effects of COVID-19 on people over 60 years of age in China and found that 37.1% experienced depression and anxiety, with gender differences in emotional responses to the pandemic (22). Moreover, in late-middle-aged adults, an inverse relationship was found between age and mental health symptoms (23). However, a comprehensive profile of the mental health status of late-middle-aged individuals during the COVID-19 pandemic does not exist. The present study evaluated mental health outcomes among late-middle-aged adults during the COVID-19 pandemic by quantifying the magnitude of depression, anxiety, insomnia, and acute stress symptoms and analyzing potential risk factors that are associated with these mental health symptoms.

## Methods

### Participants

The study was approved by the ethics committee of Peking University Sixth Hospital (Institute of Mental Health). Written informed consent was received online before the respondents began the questionnaire. This study follows the American Association for Public Opinion Research (AAPOR) reporting guidelines and the Strengthening the Reporting of Observational Studies in Epidemiology (STORBE) guidelines.

This cross-sectional, web-based study was conducted between February 28 and March 11 of 2020, during which the COVID-19 pandemic in China had stabilized and the cumulative number of confirmed cases reached a peak. A self-designed survey was released through the Joybuy web portal (http://www.jd.com/), a large commerce and information service platform with 0.44 billion active users in China by 2020. Among the 56,932 participants who provided informed consent and completed the questionnaire, 3,740 who were ≥ 50 years old completed all the survey questions. Ten participants who were infected with COVID-19 were excluded. Finally, a total of 3,730 late-middle-aged adults were included in the analyses.

### Covariates and Outcomes

The survey lasted ~15 min and consisted of four parts that gathered information about demographic variables, asked epidemic-related questions, evaluated isolation conditions and social attitudes, and used standardized mental health-related scales. All questions in the questionnaire were introduced elsewhere (24).

The covariates used in this paper could be briefly categorized into the following five parts: (1) demographic characteristics, e.g., gender, living area, level of education, marital status, and monthly family income; (2) medical conditions, e.g., history of chronic diseases, history of psychiatric disorders, and family history of psychiatric disorders; (3) epidemic-related factors, e.g., participation in frontline work, family members or friends who were infected, family members or friends participating in frontline work, residence in Hubei Province, status of work or school resumption, and risk of exposure to patients due to occupational reasons; (4) experience with public health interventions, e.g., community control, traffic restrictions, and quarantine; and (5) concern and understanding of the COVID-19 pandemic. The levels of concern about and understanding of the COVID-19 pandemic were assessed using visual analog scales that ranged from 0 to 10, in which 0 indicated no concern or understanding and 10 indicated high concern about or understanding of the COVID-19 pandemic. The levels of concern about the COVID-19 pandemic were divided into two categories: scores > 5 were defined as highly concerned about the COVID-19 pandemic, and scores ≤ 5 were defined as not highly concerned about the COVID-19 pandemic.

The main mental health outcomes were depression, anxiety, insomnia, and acute stress symptoms, which were assessed in the fourth part of the survey using Chinese versions of the 9-item Patient Health Questionnaire (PHQ-9) (25), the 7-item Generalized Anxiety Disorder Scale (GAD-7) (26), the Insomnia Severity Index (ISI) (27), and the Acute Stress Disorder Scale (ASDS) (28). Participants were classified as endorsing the corresponding symptoms according to the following cut-offs: PHQ-9 (normal [0–4], mild [5–9], moderate [10–14], and severe [15–21] depression), GAD-7 (normal [0–4], mild [5–9], moderate [10–14], and severe [15–21] anxiety), ISI (normal [0–7], subthreshold [8–14], moderate [15–21], and severe [22–28] insomnia), and ASDS (acute stress symptoms [dissociative cluster score ≥ 9 and cumulative re-experiencing, avoidance, and arousal cluster scores ≥ 28]). All measures were validated for use in Chinese populations (26, 29, 30). Based on values established in the literature (24, 31), cut-off scores of 5 for the PHQ-9, 5 for the GAD-7, and 8 for the ISI were adopted to detect depression, anxiety, and insomnia symptoms, respectively.

### Statistical Analysis

Descriptive statistics were used to analyze demographic characteristics and pandemic-related information. The prevalence of mild and moderate-to-severe depression, anxiety, insomnia, and acute stress symptoms are reported as percentages of cases in different populations among all and quarantined late-middle aged adults. χ^2^ tests were used to compare the prevalence of different mental health symptoms in stratified populations.

Respondents with missing values were removed from the multivariate logistic regression analysis. Multivariate logistic regression analysis was performed to calculate the adjusted odds ratios (AORs) and 95% confidence intervals (CIs) of the risk of mental health symptoms among all and quarantined late-middle-aged adults after adjusting for potential confounders, including demographic characteristics, medical conditions, epidemic-related factors, experience with public health interventions, and concern about and understanding of the COVID-19 pandemic. Analyses were conducted using SPSS 22 software. Statistical significance was set at *p* < 0.05, and all tests were two-tailed.

## Results

### Demographic Characteristics

Table 1 shows the demographic characteristics of the 3,730 participants. The mean age of the sample was 54.44 ± 5.99 years. Of all participants, the majority were female (54.3%), married (91.9%), lived in urban areas (97.1%), and had a university degree or higher (67.1%). The proportions of late-middle-aged adults with a history of chronic disease, a history of psychiatric disorders, and a family history of psychiatric disorders were 24.0, 0.3, and 0.6%, respectively. Of all participants, 515 (13.8%) were frontline healthcare workers, 1,165 (31.2%) had family members or friends who were frontline workers, and 28 (0.8%) had family members or friends who were infected with COVID-19. Moreover, 3,508 (94.0%) participants were highly concerned about the COVID-19 pandemic, and 3,312 (88.8%) had a good understanding of the COVID-19 pandemic. Regarding isolation conditions, 3,435 (92.1%) participants experienced community control, 2,489 (66.7%) experienced traffic restrictions, and 737 (19.8%) had been quarantined.

**Table 1:** Demographic characteristics and pandemic-related information among late-middle-aged participants.

**Factor**	**Participants, no. (%)**
Overall	3,730 (100.0)
**Gender**	
Male	1,704 (45.7)
Female	2,026 (54.3)
**Living area**	
Urban	3,623 (97.1)
Rural	107 (2.9)
**Level of education**	
Less than college	1,229 (32.9)
College degree or higher	2,501 (67.1)
**Marital status**	
Married	3,428 (91.9)
Unmarried	302 (8.1)
**Monthly family income**, ¥^a^	
0–4,999	888 (23.8)
5,000–11,999	1,742 (46.7)
≥ 12,000	1,100 (29.5)
**Region**	
Eastern	1,494 (40.1)
Northern	918 (24.6)
Northwest	114 (3.1)
Northeast	377 (10.1)
Central	273 (7.3)
Southern	337 (9.0)
Southwest	216 (5.8)
Missing	1 (0.0)
**History of chronic disease**	
Yes	896 (24.0)
No	2,712 (72.7)
Unknown	122 (3.3)
**History of psychiatric disorders**	
Yes	11 (0.3)
No	3,690 (98.9)
Unknown	29 (0.8)
**Family history of psychiatric disorders**	
Yes	23 (0.6)
No	3,672 (98.4)
Unknown	35 (0.9)
**Are you a frontline worker?**	
Yes	515 (13.8)
No	3,215 (86.2)
**Have any of your family members or friends been infected with COVID-19?**	
Yes	28 (0.8)
No	3,702 (99.2)
**Are any of your family members or friends frontline workers?**	
Yes	1,165 (31.2)
No	2,565 (68.8)
**Are you in Hubei Province now?**	
Yes	147 (3.9)
No	3,583 (96.1)
**Are you highly concerned about the COVID-19 pandemic?**	
Yes	3,508 (94.0)
No	222 (6.0)
**Do you have a good understanding of the COVID-19 pandemic?**	
Yes	3,312 (88.8)
No	418 (11.2)
**Are you back to work now?**	
Absent from work	1,035 (27.7)
Always at work	676 (18.1)
Not back to work	781 (20.9)
Back to work	1,238 (33.2)
**Are you likely to be exposed to other people at work?**	
Exposed to patients who are infected with COVID-19	121 (3.2)
Exposed to patients with other diseases	79 (2.1)
Exposed to general people	973 (26.1)
Not at work, work at home, or without exposure to people at work	2,334 (62.6)
Missing values	223 (6.0)
**Do you live in a community that restricts people's access?**	
Yes	3,435 (92.1)
No	295 (7.9)
**Were there any traffic restrictions in your area during the pandemic?**	
Yes	2,489 (66.7)
No	1,241 (33.3)
**Have you ever experienced quarantine?**	
Yes	737 (19.8)
No	2,993 (80.2)

*COVID-19, coronavirus disease 2019.*

a *1 ¥ = USD$0.14*.

### Prevalence of Mental Health Symptoms in Late-Middle-Aged Adults

A total of 761 (20.4%) respondents reported depression symptoms, including 473 (12.7%) with mild depressive symptoms and 288 (7.7%) with moderate-to-severe depressive symptoms. A total of 1,011 (27.1%) respondents had anxiety symptoms, including 688 (18.4%) with mild anxiety and 323 (8.7%) with moderate-to-severe anxiety. A total of 1,027 (27.5%) respondents had insomnia symptoms, including 820 (22.0%) with mild insomnia symptoms and 207 (5.8%) with moderate-to-severe insomnia symptoms. A total of 791 (21.2%) respondents reported acute stress symptoms.

The prevalence of depression, anxiety, insomnia, and acute stress symptoms was high among the following groups of participants: (1) participants with a history of psychiatric disorders (depression, 72.7%; anxiety, 72.7%; insomnia, 54.5%; acute stress, 54.5%); (2) participants who experienced traffic restrictions (depression, 22.3%; anxiety, 29.0%; insomnia, 29.1%; acute stress, 22.5%); and (3) participants who had been quarantined (depression, 26.1%; anxiety, 33.2%; insomnia, 32.4%; acute stress, 25.9%). Individuals with a good understanding of the COVID-19 epidemic had a low prevalence of depression (19.1%), anxiety (26.1%), insomnia (26.6%), and acute stress (20.1%) symptoms. The following groups of late-middle-aged adults had a high prevalence of depression, anxiety, and acute stress symptoms: (1) those with a low income (0–4,999 yuan/month: depression [22.6%], anxiety [30.7%], acute stress [23.8%]; 5,000–11,999 yuan/month: depression [21.6%], anxiety [28.1%], acute stress [22.5%]); (2) residents of Hubei Province (depression, 29.9%; anxiety, 42.2%; acute stress, 29.9%); and (3) those who were likely to be exposed to patients who were infected with COVID-19 at work (depression, 38.0%; anxiety, 45.5%; acute stress, 35.5%). Additional details regarding the prevalence of mental health symptoms in the different populations are presented in Table 2. Additionally, the prevalence of mental health symptoms in the quarantined populations is presented in Supplementary Table 1.

**Table 2:** Categories of severity of anxiety, depression, insomnia, and acute stress in late-middle-aged adults stratified by pandemic-related factors.

	**Depression^a^**				**Anxiety^b^**				**Insomnia^c^**				**Acute stress^d^**		
	**Participants, no. (%)**				**Participants, no. (%)**				**Participants, no. (%)**				**Participants, no. (%)**		
**Variables**	**Normal**	**Mild**	**Moderate to severe**	***p*^e^**	**Normal**	**Mild**	**Moderate to severe**	***p*^e^**	**Normal**	**Mild**	**Moderate to severe**	***p*^e^**	**Normal**	**Stressed**	***p*^e^**
**Overall**	2,969 (79.6)	473 (12.7)	288 (7.7)		2,719 (72.9)	688 (18.4)	323 (8.7)		2,703 (72.5)	820 (22.0)	207 (5.5)		29,39 (78.8)	791 (21.2)	
**Gender**				0.912				0.422				0.119			0.283
Male	1,355 (79.5)	223 (13.1)	126 (7.4)		1,253 (73.5)	305 (17.9)	146 (8.6)		1,256 (73.7)	364 (21.4)	84 (4.9)		1,356 (79.6)	348 (20.4)	
Female	1,614 (79.9)	250 (12.3)	162 (8.0)		1,466 (72.4)	383 (18.9)	177 (8.7)		1,447 (71.4)	456 (22.5)	123 (6.1)		1,583 (78.1)	443 (21.9)	
**Living area**				0.081				0.123				0.224			0.427
Urban	2,891 (79.8)	456 (12.6)	276 (7.6)		2,648 (73.1)	663 (18.3)	312 (8.6)		2,631 (72.6)	796 (22.0)	196 (5.4)		2,858 (78.9)	765 (21.1)	
Rural	78 (72.9)	17 (15.9)	12 (11.2)		71 (66.4)	25 (23.4)	11 (10.3)		72 (67.3)	24 (22.4)	11 (10.3)		81 (75.7)	26 (24.3)	
Level of education				0.136				0.086				0.839			0.587
Less than college	961 (78.2)	160 (13.0)	108 (8.8)		874 (71.1)	235 (19.1)	120 (9.8)		888 (72.3)	269 (21.9)	72 (5.9)		962 (78.3)	267 (21.7)	
College degree or higher	2,008 (80.3)	313 (12.5)	180 (7.2)		1,845 (73.8)	453 (18.1)	203 (8.1)		1,815 (72.6)	551 (22.0)	135 (5.4)		1,977 (79.0)	524 (21.0)	
**Marital status**				0.122				0.407				0.011			0.878
Married	2,739 (79.9)	428 (12.5)	261 (7.6)		2,505 (73.1)	632 (18.4)	291 (8.5)		2,503 (73.0)	739 (21.6)	186 (5.4)		2,700 (78.8)	728 (21.2)	
Unmarried	230 (76.2)	45 (14.9)	27 (8.9)		214 (70.9)	56 (18.5)	32 (10.6)		200 (66.2)	81 (26.8)	21 (7.0)		239 (79.1)	63 (20.9)	
**Monthly family income**, ¥**^f^**				0.001				<0.001				0.278			< 0.001
0–4,999	687 (77.4)	123 (13.9)	78 (8.8)		615 (69.3)	181 (20.4)	92 (10.4)		638 (71.8)	200 (22.5)	50 (5.6)		677 (76.2)	211 (23.8)	
5,000–11,999	1,366 (78.4)	226 (13.0)	150 (8.6)		1,253 (71.9)	329 (18.9)	160 (9.2)		1,248 (71.6)	396 (22.7)	98 (5.6)		1,350 (77.5)	392 (22.5)	
≥ 12,000	916 (83.3)	124 (11.3)	60 (5.5)		851 (77.4)	178 (16.2)	71 (6.5)		817 (74.3)	224 (20.4)	59 (5.4)		912 (82.9)	188 (17.1)	
**History of chronic disease**				0.178				0.021				<0.001			0.132
Yes	696 (77.7)	124 (13.8)	76 (8.5)		648 (72.3)	166 (18.5)	82 (9.2)		598 (66.7)	232 (25.9)	66 (7.4)		692 (77.2)	204 (22.8)	
No	2,179 (80.3)	331 (12.2)	202 (7.4)		1,995 (73.6)	489 (18.0)	228 (8.4)		2,028 (74.8)	558 (20.6)	126 (4.6)		2,157 (79.5)	555 (20.5)	
Unknown	94 (77.0)	18 (14.8)	10 (8.2)		76 (62.3)	33 (27.0)	13 (10.7)		77 (63.1)	30 (24.6)	15 (12.3)		90 (73.8)	32 (26.2)	
**History of psychiatric disorders**				< 0.001				< 0.001				< 0.001			0.002
Yes	3 (27.3)	4 (36.4)	4 (36.4)		3 (27.3)	4 (36.4)	4 (36.4)		5 (45.5)	2 (18.2)	4 (36.4)		5 (45.5)	6 (54.5)	
No	2,946 (79.8)	466 (12.6)	278 (7.5)		2,700 (73.2)	679 (18.4)	311 (8.4)		2,685 (72.8)	808 (21.9)	197 (5.3)		2,916 (79.0)	774 (21.0)	
Unknown	20 (69.0)	3 (10.3)	6 (20.7)		16 (55.2)	5 (17.2)	8 (27.6)		13 (44.8)	10 (34.5)	6 (20.7)		18 (62.1)	11 (37.9)	
**Family history of psychiatric disorders**				0.059				0.022				0.240			0.511
Yes	16 (69.6)	4 (17.4)	3 (13.0)		13 (56.5)	9 (39.1)	1 (4.3)		16 (69.6)	4 (17.4)	3 (13.0)		19 (82.6)	4 (17.4)	
No	2,930 (79.8)	466 (12.7)	276 (7.5)		2,686 (73.1)	672 (18.3)	314 (8.6)		2,666 (72.6)	807 (22.0)	199 (5.4)		2,895 (78.8)	777 (21.2)	
Unknown	23 (65.7)	3 (8.6)	9 (25.7)		20 (57.1)	7 (20.0)	8 (22.9)		21 (60.0)	9 (25.7)	5 (14.3)		25 (71.4)	10 (28.6)	
**Are you a frontline worker?**				0.014				0.009				0.041			0.086
Yes	389 (75.5)	70 (13.6)	56 (10.9)		351 (68.2)	105 (20.4)	59 (11.5)		354 (68.7)	129 (25.0)	32 (6.2)		391 (75.9)	124 (24.1)	
No	2,580 (80.2)	403 (12.5)	232 (7.2)		2,368 (73.7)	583 (18.1)	264 (8.2)		2,349 (73.1)	691 (21.5)	175 (5.4)		2,548 (79.3)	667 (20.7)	
**Have any of your family members or friends been infected with COVID-19?**				0.122				0.304				0.162			0.019
Yes	19 (67.9)	3 (10.7)	6 (21.4)		18 (64.3)	5 (17.9)	5 (17.9)		17 (60.7)	8 (28.6)	3 (10.7)		17 (60.7)	11 (39.3)	
No	2,950 (79.7)	470 (12.7)	282 (7.6)		2,701 (73.0)	683 (18.4)	318 (8.6)		2,686 (72.6)	812 (21.9)	204 (5.5)		2,922 (78.9)	780 (21.1)	
**Are any of your family members or friends frontline workers?**				0.641				0.077				0.017			0.439
Yes	922 (79.1)	147 (12.6)	96 (8.2)		827 (71.0)	226 (19.4)	112 (9.6)		814 (69.9)	288 (24.7)	63 (5.4)		909 (78.0)	256 (22.0)	
No	2,047 (79.8)	326 (12.7)	192 (7.5)		1,892 (73.8)	462 (18.0)	211 (8.2)		1,889 (73.6)	532 (20.7)	144 (5.6)		2,030 (79.1)	535 (20.9)	
**Are you in Hubei Province now?**				0.003				< 0.001				0.108			0.008
Yes	103 (70.1)	24 (16.3)	20 (13.6)		85 (57.8)	39 (26.5)	23 (15.6)		98 (66.7)	40 (27.2)	9 (6.1)		103 (70.1)	44 (29.9)	
No	2,866 (80.0)	449 (12.5)	268 (7.5)		2,634 (73.5)	649 (18.1)	300 (8.4)		2,605 (72.7)	780 (21.8)	198 (5.5)		2,836 (79.2)	747 (20.8)	
**Are you back to work now?**				0.187				0.353				0.343			0.342
Absent from work	828 (80.0)	117 (11.3)	90 (8.7)		764 (73.8)	176 (17.0)	95 (9.2)		745 (72.0)	222 (21.4)	68 (6.6)		803 (77.6)	232 (22.4)	
Always at work	527 (78.0)	96 (14.2)	53 (7.8)		486 (71.9)	128 (18.9)	62 (9.2)		476 (70.4)	160 (23.7)	40 (5.9)		536 (79.3)	140 (20.7)	
Not back to work	608 (77.8)	110 (14.1)	63 (8.1)		553 (70.8)	155 (19.8)	73 (9.3)		564 (72.2)	178 (22.8)	39 (5.0)		606 (77.6)	175 (22.4)	
Back to work	1,006 (81.3)	150 (12.1)	82 (6.6)		916 (74.0)	229 (18.5)	93 (7.5)		918 (74.2)	260 (21.0)	60 (4.8)		994 (80.3)	244 (19.7)	
**Are you likely to be exposed to other people at work?**				< 0.001				< 0.001				0.504			< 0.001
Exposed to patients infected with COVID-19	75 (62.0)	26 (21.5)	20 (16.5)		66 (54.5)	34 (28.1)	21 (17.4)		80 (66.1)	31 (25.6)	10 (8.3)		78 (64.5)	43 (35.5)	
Exposed to patients with other diseases	63 (79.7)	9 (11.4)	7 (8.9)		60 (75.9)	14 (17.7)	5 (6.3)		57 (72.2)	20 (25.3)	2 (2.5)		63 (79.7)	16 (20.3)	
Exposed to general people	796 (81.8)	115 (11.8)	62 (6.4)		723 (74.3)	178 (18.3)	72 (7.4)		714 (73.4)	217 (22.3)	42 (4.3)		799 (82.1)	174 (17.9)	
Not at work, work at home, or without exposure to people at work	1,884 (80.7)	296 (12.7)	154 (6.6)		1,731 (74.2)	423 (18.1)	180 (7.7)		1,706 (73.1)	493 (21.1)	135 (5.8)		1,853 (79.4)	481 (20.6)	
**Do you live in a community that restricts people's access?**				0.435				0.277				0.327			0.704
Yes	2,729 (79.4)	445 (13.0)	261 (7.6)		2,496 (72.7)	640 (18.6)	299 (8.7)		2,482 (72.3)	759 (22.1)	194 (5.6)		2,704 (78.7)	731 (21.3)	
No	240 (81.4)	28 (9.5)	27 (9.2)		223 (75.6)	48 (16.3)	24 (8.1)		221 (74.9)	61 (20.7)	13 (4.4)		235 (79.7)	60 (20.3)	
**Were there any traffic restrictions in your area during the pandemic?**				< 0.001				< 0.001				0.003			0.006
Yes	1,935 (77.7)	351 (14.1)	203 (8.2)		1,767 (71.0)	482 (19.4)	240 (9.6)		1,765 (70.9)	580 (23.3)	144 (5.8)		1,929 (77.5)	560 (22.5)	
No	1,034 (83.3)	122 (9.8)	85 (6.8)		952 (76.7)	206 (16.6)	83 (6.7)		938 (75.6)	240 (19.3)	63 (5.1)		1,010 (81.4)	231 (18.6)	
**Have you ever experienced quarantine?**				< 0.001				<0.001				0.001			< 0.001
Yes	545 (73.9)	108 (14.7)	84 (11.4)		492 (66.8)	148 (20.1)	97 (13.2)		498 (67.6)	181 (24.6)	58 (7.9)		546 (74.1)	191 (25.9)	
No	2,424 (81.0)	365 (12.2)	204 (6.8)		2,227 (74.4)	540 (18.0)	226 (7.6)		2,205 (73.7)	639 (21.3)	149 (5.0)		2,393 (80.0)	600 (20.0)	
**Are you highly concerned about the COVID-19 pandemic?**				0.004				0.364				0.363			0.004
Yes	2,809 (80.1)	437 (12.5)	262 (7.5)		2,563 (73.1)	645 (18.4)	300 (8.6)		2,548 (72.6)	768 (21.9)	192 (5.5)		2,781 (79.3)	727 (20.7)	
No	160 (72.1)	36 (16.2)	26 (11.7)		156 (70.3)	43 (19.4)	23 (10.4)		155 (69.8)	52 (23.4)	15 (6.8)		158 (71.2)	64 (28.8)	
**Do you have a good understanding of the COVID-19 pandemic?**				< 0.001				< 0.001				< 0.001			< 0.001
Yes	2,681 (80.9)	398 (12.0)	233 (7.0)		2,449 (73.9)	593 (17.9)	270 (8.2)		2,431 (73.4)	705 (21.3)	176 (5.3)		2,645 (79.9)	667 (20.1)	
No	288 (68.9)	75 (17.9)	55 (13.2)		270 (64.6)	95 (22.7)	53 (12.7)		272 (65.1)	115 (27.5)	31 (7.4)		294 (70.3)	124 (29.7)	

*COVID-19, coronavirus disease 2019*.

a *Scores of 5–9 on the Patient Health Questionnaire−9 were defined as mild depression, and scores of ≥ 10 were defined as moderate-to-severe depression*.

b *Scores of 5–9 on the Generalized Anxiety Disorder−7 were defined as mild anxiety, and scores of ≥ 10 were defined as moderate-to-severe anxiety*.

c * cores of 8–14 on the Insomnia Severity Index were defined as subthreshold insomnia, and scores of ≥ 15 were defined as moderate-to-severe insomnia*.

d *Acute stress symptoms were defined as having an Acute Stress Disorder Scale dissociative cluster score of ≥ 9 and cumulative re-experiencing, avoidance, and arousal cluster scores of ≥ 28*.

e *χ^2^ tests were used to compare the prevalence of mild-to-severe mental health symptoms in different populations*.

f *1 ¥ = USD$0.14*.

### Factors Associated With Mental Health Symptoms in Late-Middle-Aged Adults

A total of 223 participants (6.0%) were excluded from the regression analysis because of missing data. Several personal factors were associated with mental health symptoms. Male participants (AOR = 0.83, 95% CI = 0.70−0.97, *p* = 0.020) and married individuals (AOR = 0.76, 95% CI = 0.58–1.00, *p* = 0.046) had a lower risk of insomnia symptoms. Compared with participants who had a family income ≥ 12,000 yuan/month, late-middle-aged adults with low income were more susceptible to the following mental health symptoms: (1) depression (0–4,999 yuan/month: AOR = 1.35, 95% CI = 1.04–1.76, *p* = 0.026; 5,000–11,999 yuan/month: AOR = 1.37, 95% CI = 1.11–1.69, *p* = 0.004); (2) anxiety (0–4,999 yuan/month: AOR = 1.48, 95% CI = 1.17–1.87, *p* = 0.001; 5,000–11,999 yuan/month: AOR = 1.35, 95% CI = 1.12–1.63, *p* = 0.002); and (3) acute stress (0–4,999 yuan/month: AOR = 1.44; 95% CI = 1.11–1.87, *p* = 0.006; 5,000–11,999 yuan/month: AOR = 1.40, 95% CI = 1.14–1.72, *p* = 0.001). Additionally, associations were found between the following factors: (1) a history of chronic disease and insomnia (AOR = 1.51, 95% CI = 1.27–1.80, *p* < 0.001); (2) a history of psychiatric disorders and depression (AOR = 6.56, 95% CI = 1.62–26.56, *p* = 0.008); (3) a history of psychiatric disorders and anxiety (AOR = 5.01, 95% CI = 1.25–20.12, *p* = 0.023); and (4) a family history of mental disorders and anxiety (AOR = 2.96, 95% CI = 1.22–7.21, *p* = 0.017).

Participants who were likely to be exposed to patients who were infected with COVID-19 at work had a higher mental health risk than participants without a risk of exposure to patients who were infected with COVID-19. The AORs were as follows: (1) 2.57 (95% CI = 1.67–3.97, *p* < 0.001) for depression; (2) 2.39 (95% CI = 1.58–3.61, *p* < 0.001) for anxiety; and (3) 2.05 (95% CI = 1.32–3.16, *p* = 0.001) for acute stress. Additionally, participants with family members or friends who were infected with COVID-19 had a higher risk of acute stress (AOR = 2.28, 95% CI = 1.00–5.20; *p* < 0.050), and Hubei residents had a higher risk of anxiety symptoms (AOR = 1.63, 95% CI = 1.11–2.40, *p* = 0.012).

Participants who were quarantined exhibited a higher risk for all mental health symptoms after adjustment (depression: AOR = 1.35, 95% CI = 1.10–1.67, *p* = 0.005; anxiety: AOR = 1.28, 95% CI = 1.06–1.55, *p* = 0.012; insomnia: AOR = 1.30, 95% CI = 1.07–1.57, *p* = 0.007; acute stress: AOR = 1.36, 95% CI = 1.11–1.67, *p* = 0.003). Participants who experienced traffic restrictions reported depressive symptoms (AOR = 1.28, 95% CI = 1.04–1.57, *p* = 0.018) when compared with participants without traffic restrictions. In quarantined late-middle-aged adults, lower family income was associated with a higher risk for developing the following mental health symptoms: (1) depression (0–4,999 yuan/month: AOR = 2.77, 95% CI = 1.55–4.95, *p* = 0.001; 5,000–11,999 yuan/month: AOR = 1.92, 95% CI = 1.19–3.08, *p* = 0.008); (2) anxiety (0–4,999 yuan/month: AOR = 2.27, 95% CI = 1.35–3.84, *p* = 0.002; 5,000–11,999 yuan/month: AOR = 1.68, 95% CI = 1.10–2.56, *p* = 0.017); and (3) acute stress (0–4,999 yuan/month: AOR = 1.95, 95% CI = 1.10–3.44, p = 0.022; 5,000–11,999 yuan/month: AOR = 1.74, 95% CI = 1.10–2.75, *p* = 0.017). However, a lower education level resulted in a lower risk for depression (AOR = 0.53, 95% CI = 0.41–0.83, *p* = 0.005) and acute stress (AOR = 0.64, 95% CI = 0.41–0.98, *p* = 0.040). The detailed results of the multivariate analysis of the risk factors associated with mental health symptoms in quarantined late-middle-aged adults are shown in Supplementary Table 2.

Moreover, participants who had a good understanding of the COVID-19 pandemic were less vulnerable to depression (AOR = 0.55, 95% CI = 0.42–0.73, *p* < 0.001), anxiety (AOR = 0.65, 95% CI = 0.50–0.85, *p* = 0.002), insomnia (AOR = 0.67, 95% CI = 0.52–0.87, *p* = 0.003), and acute stress (AOR = 0.66, 95% CI = 0.50–0.88, *p* = 0.004). The detailed results of the multivariate analysis of the risk factors associated with depression, anxiety, insomnia, and acute stress symptoms in late-middle-aged adults during the COVID-19 pandemic are shown in Table 3.

**Table 3:** Multivariable regression analysis of the risk factors associated with depression, anxiety, insomnia, and acute stress symptoms in late-middle-aged adults during the COVID-19 pandemic.

	**Depression**		**Anxiety**		**Insomnia**		**Acute stress**	
**Variable**	**AOR (95% CI)**	***p***	**AOR (95% CI)**	***p***	**AOR (95% CI)**	***p***	**AOR (95% CI)**	***P***
**Gender**								
Male	0.96 (0.80–1.15)	0.642	0.86 (0.73–1.01)	0.075	0.83 (0.70–0.97)	0.020	0.86 (0.72–1.03)	0.107
Female	1 [Reference]		1 [Reference]		1 [Reference]		1 [Reference]	
**Living area**								
Urban	0.90 (0.55–1.47)	0.682	0.89 (0.56–1.39)	0.598	0.88 (0.56–1.39)	0.595	1.13 (0.68–1.89)	0.642
Rural	1 [Reference]		1 [Reference]		1 [Reference]		1 [Reference]	
**Level of education**								
Less than college	0.94 (0.77–1.16)	0.560	0.99 (0.82–1.19)	0.879	0.96 (0.80–1.15)	0.672	0.86 (0.70–1.06)	0.153
College degree or higher	1 [Reference]		1 [Reference]		1 [Reference]		1 [Reference]	
**Marital status**								
Married	0.89 (0.65–1.20)	0.434	1.02 (0.77–1.35)	0.887	0.76 (0.58–1.00)	0.046	1.15 (0.84–1.58)	0.375
Unmarried	1 [Reference]		1 [Reference]		1 [Reference]		1 [Reference]	
**Monthly family income**, ¥**^a^**								
0–4,999	1.35 (1.04–1.76)	0.026	1.48 (1.17–1.87)	0.001	1.03 (0.82–1.30)	0.800	1.44 (1.11–1.87)	0.006
5,000–11,999	1.37 (1.11–1.69)	0.004	1.35 (1.12–1.63)	0.002	1.11 (0.93–1.33)	0.253	1.40 (1.14–1.72)	0.001
≥ 12,000	1 [Reference]		1 [Reference]		1 [Reference]		1 [Reference]	
**History of chronic disease**								
Yes	1.18 (0.97–1.44)	0.103	1.06 (0.88–1.27)	0.566	1.51 (1.27–1.80)	< 0.001	1.15 (0.95–1.40)	0.161
No	1 [Reference]		1 [Reference]		1 [Reference]		1 [Reference]	
Unknown	1.19 (0.74–1.91)	0.484	1.55 (1.03–2.35)	0.036	1.64 (1.09–2.47)	0.019	1.37 (0.87–2.16)	0.168
**History of psychiatric disorders**								
Yes	6.56 (1.62–26.56)	0.008	5.01 (1.25–20.12)	0.023	3.16 (0.85–11.68)	0.085	3.21 (0.88–11.68)	0.077
No	1 [Reference]		1 [Reference]		1 [Reference]		1 [Reference]	
Unknown	0.46 (0.11–1.97)	0.298	1.04 (0.33–3.33)	0.942	2.84 (0.91–8.90)	0.073	1.71 (0.49–5.93)	0.396
**Family history of psychiatric disorders**								
Yes	2.09 (0.81–5.40)	0.130	2.96 (1.22–7.21)	0.017	0.97 (0.37–2.58)	0.956	0.99 (0.32–3.04)	0.990
No	1 [Reference]		1 [Reference]		1 [Reference]		1 [Reference]	
Unknown	1.70 (0.57–5.08)	0.346	1.45 (0.53–3.92)	0.468	0.61 (0.20–1.81)	0.369	0.62 (0.19–2.07)	0.438
**Are you a frontline worker?**								
Yes	1.21 (0.91–1.60)	0.184	1.19 (0.93–1.53)	0.174	1.15 (0.89–1.48)	0.278	1.26 (0.96–1.66)	0.102
No	1 [Reference]		1 [Reference]		1 [Reference]		1 [Reference]	
**Have any of your family members or friends been infected with COVID-19?**								
Yes	1.62 (0.69–3.83)	0.272	1.20 (0.52–2.78)	0.664	1.62 (0.72–3.65)	0.241	2.28 (1.00–5.20)	< 0.050
No	1 [Reference]		1 [Reference]		1 [Reference]		1 [Reference]	
**Are any of your family members or friends frontline workers?**								
Yes	0.93 (0.76–1.13)	0.481	1.04 (0.87–1.24)	0.685	1.13 (0.95–1.34)	0.181	0.98 (0.81–1.19)	0.813
No	1 [Reference]		1 [Reference]		1 [Reference]		1 [Reference]	
**Are you in Hubei Province now?**								
Yes	1.37 (0.90–2.08)	0.141	1.63 (1.11–2.40)	0.012	1.02 (0.68–1.53)	0.918	1.28 (0.84–1.95)	0.248
No	1 [Reference]		1 [Reference]		1 [Reference]		1 [Reference]	
**Are you back to work now?**								
Absent from work	0.92 (0.68–1.23)	0.560	0.87 (0.67–1.13)	0.299	0.98 (0.75–1.26)	0.848	0.95 (0.72–1.26)	0.738
Always at work	1.11 (0.87–1.43)	0.402	1.00 (0.80–1.26)	0.968	1.15 (0.92–1.44)	0.216	0.94 (0.73–1.21)	0.641
Not back to work	1.12 (0.83–1.50)	0.453	1.07 (0.82–1.39)	0.636	0.99 (0.76–1.29)	0.928	0.92 (0.69–1.23)	0.577
Back to work	1 [Reference]		1 [Reference]		1 [Reference]		1 [Reference]	
**Are you likely to be exposed to other people at work?**								
Exposed to patients infected with COVID−19	2.57 (1.67–3.97)	< 0.001	2.39 (1.58–3.61)	< 0.001	1.39 (0.91–2.14)	0.130	2.05 (1.32–3.16)	0.001
Exposed to patients with other diseases	1.06 (0.58–1.94)	0.853	0.93 (0.53–1.64)	0.802	1.02 (0.59–1.74)	0.954	0.96 (0.53–1.75)	0.889
Exposed to general people	0.99 (0.76–1.29)	0.925	1.05 (0.83–1.33)	0.696	1.02 (0.81–1.29)	0.869	0.86 (0.66–1.12)	0.275
Not at work, work at home, or without exposure to people at work	1 [Reference]		1 [Reference]		1 [Reference]		1 [Reference]	
**Do you live in a community that restricts people's access?**								
Yes	1.12 (0.78–1.61)	0.533	1.16 (0.84–1.60)	0.363	1.05 (0.77–1.44)	0.739	1.06 (0.75–1.50)	0.735
No	1 [Reference]		1 [Reference]		1 [Reference]		1 [Reference]	
**Were there any traffic restrictions in your area during the pandemic?**								
Yes	1.28 (1.04–1.57)	0.018	1.16 (0.97–1.38)	0.116	1.18 (0.99–1.41)	0.066	1.17 (0.96–1.43)	0.114
No	1 [Reference]		1 [Reference]		1 [Reference]		1 [Reference]	
**Have you ever experienced quarantine?**								
Yes	1.35 (1.10–1.67)	0.005	1.28 (1.06–1.55)	0.012	1.30 (1.07–1.57)	0.007	1.36 (1.11–1.67)	0.003
No	1 [Reference]		1 [Reference]		1 [Reference]		1 [Reference]	
**Are you highly concerned about the COVID-19 pandemic?**								
Yes	1.04 (0.70–1.54)	0.858	1.27 (0.87–1.85)	0.220	1.12 (0.78–1.62)	0.540	0.86 (0.59–1.26)	0.437
No	1 [Reference]		1 [Reference]		1 [Reference]		1 [Reference]	
**Do you have a good understanding of the COVID-19 pandemic?**								
Yes	0.55 (0.42–0.73)	< 0.001	0.65 (0.50–0.85)	0.002	0.67 (0.52–0.87)	0.003	0.66 (0.50–0.88)	0.004
No	1 [Reference]		1 [Reference]		1 [Reference]		1 [Reference]	

*AOR, adjusted odds ratio; COVID-19, coronavirus disease 2019*.

a *1 ¥ = USD$0.14*.

## Discussion

This cross-sectional survey enrolled 3,730 respondents and determined the prevalence of mental health symptoms among late-middle-aged adults in China during the COVID-19 pandemic. Overall, 20.4, 27.1, 27.5, and 21.2% of late-middle-aged adults reported depression, anxiety, insomnia, and acute stress symptoms, respectively. After controlling for confounding factors, including demographic characteristics and pandemic-related factors, quarantine experience and the level of understanding of the COVID-19 pandemic were associated with all four mental health outcomes. Participants with a low income and who had COVID-19 exposure risk at work had a remarkably high risk of depression, anxiety, and acute stress symptoms. These findings may help understanding about the impact of the COVID-19 pandemic on mental health in late-middle-aged adults and provide information for stratified psychological prevention and intervention strategies.

Previous studies have mainly focused on young and middle-aged adults (32–34). The mental health status of late-middle-aged adults has been relatively understudied. The present study found that approximately one-fifth (20.4%) to one-quarter (27.5%) of late-middle-aged adults experienced mild-to-severe mental health symptoms, including anxiety, depression, insomnia, and acute stress, during the COVID-19 pandemic in China. The prevalence of anxiety and acute stress in the present study was comparable to another study in late-middle-aged Australian adults, but the prevalence of depressive symptoms in Australia was higher than in the present study, which may be attributable to cultural differences and different measures (35). A previous study of 1,556 adults aged ≥ 60 years reported that 37.1% had anxiety or depression symptoms during the COVID-19 crisis (22). We found that 30.1% of late-middle-aged adults had depression or anxiety symptoms (17.4% of the participants had both depression and anxiety, 3.0% participants had depression only, and 9.7% participants had anxiety only), which was similar to but slightly lower than previous findings. These differences may have resulted from the distinct study design, different demographic characteristics of the population, and the time of data collection during the COVID-19 pandemic. Compared with young individuals from the same sample, the prevalence of mental health symptoms was lower in late-middle-aged adults (24). Older adults had a high prevalence of a history of chronic disease and low monthly family income, whereas more young individuals had family members or friends who were infected with COVID-19, had been quarantined, and were more likely to be exposed to patients who were infected with COVID-19, which increased the risk of mental health symptoms during the pandemic (24). We speculate that resilience is important when late-middle-aged adults confront the COVID-19 pandemic (36, 37). Further studies are needed to investigate the prevalence of mental health symptoms in late-middle-aged adults and to compare mental health outcomes in different populations during this public health emergency.

The present study identified several factors that were strongly associated with mental health symptoms in late-middle-aged adults during the COVID-19 pandemic. Notably, individuals with quarantine experience had a higher risk of all reported mental health symptoms. Moreover, low family income was associated with several mental health symptoms in quarantined participants. Quarantine has emerged as an effective public health measure to restrain the spread of COVID-19 infection, but it can hamper access to basic supplies, disrupt information flow, and increase both fear and anxiety (38, 39). Additionally, quarantine experience also leads to social isolation and a sense of loneliness, especially for geriatric populations who may be less comfortable using online tools (12, 13, 15, 40). Increases in proinflammatory immune responses and decreases in antiviral immune responses may be involved in the mechanism that underlies the impact of quarantine experience on mental health outcomes (13, 41). Several strategies could be developed to cope with the negative affect caused by quarantine. First, the quarantine period should be as short as possible because longer quarantine periods are associated with poorer psychological outcomes (38). Second, adequate supplies need to be provided to late-middle-aged adults, especially those who are impoverished. Third, social connections need to be enhanced, such as regular phone calls and suitable online applications (12, 42). Fourth, regular physical activity and mindfulness practices should be implemented during the pandemic (40, 43).

Understanding COVID-19-related information and being cognizant of exposure risk at work were two other important risk factors for mental health symptoms in late-middle-aged adults. Similar to previous findings, most of the participants had a good understanding and knowledge of the pandemic (44). Our findings showed that a good understanding of the COVID-19 pandemic could help relieve mental health symptoms, including depression, anxiety, insomnia, and acute stress. This indicates the need to disseminate pandemic-related information to the late-middle-aged population during the pandemic (45, 46). Additionally, compared with participants who did not have a risk of exposure to COVID-19 patients at work, late-middle-aged adults who were potentially exposed to COVID-19 patients at work had a higher risk of developing depression, anxiety, and acute stress. This finding is consistent with previous studies of the general population, healthcare workers, and technical staff (24, 47, 48), thus demonstrating that providing more personal protective equipment for people with jobs that have a high exposure risk can improve well-being during the COVID-19 pandemic.

Some demographic characteristics, especially income level, were associated with mental health symptoms. Low income was associated with a higher risk of depression, anxiety, and acute stress symptoms. Poverty leads to an increase in the prevalence of mental health symptoms (49, 50). Past experience suggests that the consequences of economic downturns can be devastating for the elderly (51). During the pandemic, income losses can destroy work plans, increase life burdens, and render people more susceptible to mental illness (51, 52). Therefore, late-middle-aged adults with a low family income should receive more access to social support. In the present study, a history of chronic disease was not a significant risk factor for depression, anxiety, or acute stress symptoms. Late-middle-aged adults with chronic disease only exhibited insomnia symptoms, which contradicts a survey in the Spanish population aged ≥ 60 years that reported a higher prevalence of depressive and anxiety symptoms in individuals with chronic disease (53). These disparate findings can be partially explained by differences in age and living area. Most of the participants in the present study were relatively young and lived in urban areas. Therefore, they may have fewer comorbidities and can receive medical assistance more easily. This indicates that sufficient medical care, including mental health services, is necessary for this population.

The present study has limitations. First, selection bias may be unavoidable because of the use of an online social media application to recruit participants. The survey was conducted among internet users who were highly educated and more concerned about the pandemic; thus, the representativeness of the sample might be limited. Second, all the variables were only self-reported and not confirmed with validated tools, which may inflate the relationship between those factors and mental health symptoms. Third, this was a cross-sectional study that lacked a longitudinal follow-up. Dynamic changes in mental health symptoms among late-middle-aged adults during different phases of the COVID-19 pandemic are unknown. Long-term psychological implications in this population should be investigated further.

## Conclusions

In conclusion, late-middle-aged adults had a relatively high prevalence of depression, anxiety, insomnia, and acute stress symptoms during the COVID-19 pandemic in this survey. Factors such as quarantine experience, the level of understanding of the COVID-19 pandemic, risk of exposure to patients with COVID-19 at work, and economic status were associated with mental health symptoms in late-middle-aged adults. These findings indicate that mental health symptoms are common among late-middle-aged adults during the COVID-19 pandemic. Stratified interventions to promote well-being in late-middle-aged adults should be implemented during the pandemic. Future studies are needed to explore the long-term effects of COVID-19 on mental health in late-middle-aged adults.

## Data Availability Statement

The raw data supporting the conclusions of this article will be made available by the authors, without undue reservation.

## Ethics Statement

The studies involving human participants were reviewed and approved by ethics committee of Peking University Sixth Hospital. The patients/participants provided their written informed consent to participate in this study.

## Author Contributions

Y-BZ, LS, Z-AL, J-YQ, X-LH, Y-PB, JS, and LLu conceived and designed the framework of this study. LS, Z-AL, J-YQ, and X-LH collected data. Y-BZ, LS, Z-AL, LLiu, Y-HW, Q-DL, and ZW executed the statistical analyses. Y-BZ and LS drafted the manuscript. KY, WY, YH, X-YS, Y-PB, JS, and LLu revised the manuscript. All authors read and approved the final manuscript.

## Conflict of Interest

The authors declare that the research was conducted in the absence of any commercial or financial relationships that could be construed as a potential conflict of interest.

## References

[1] Meo SA Alhowikan AM Al-Khlaiwi T Meo IM Halepoto DM Iqbal M. Novel coronavirus 2019-nCoV: prevalence, biological and clinical characteristics comparison with SARS-CoV and MERS-CoV. Eur Rev Med Pharmacol Sci. (2020) 24:2012–9. 10.26355/eurrev_202002_20379 32141570

[2] WHO. Coronavirus Disease (COVID-19) Pandemic (2020). Available online at: https://www.who.int/emergencies/diseases/novel-coronavirus-2019. (accessed December 19, 2020).

[3] Zwald ML Lin W Sondermeyer Cooksey GL Weiss C Suarez A Fischer M. Rapid sentinel surveillance for covid 19 Santa Clara County, California, March 2020. MMWR Morb Mortal Wkly Rep. (2020) 69:419–21. 10.15585/mmwr.mm6914e3 32271724 PMC7147906

[4] Ebrahim SH Ahmed QA Gozzer E Schlagenhauf P Memish ZA. COVID-19 and community mitigation strategies in a pandemic. BMJ. (2020) 368:m1066. 10.1136/bmj.m1066 32184233

[5] Bao Y Sun Y Meng S Shi J Lu L. 2019-nCoV epidemic: address mental health care to empower society. Lancet. (2020) 395:e37–8. 10.1016/S0140-6736(20)30309-3 32043982 PMC7133594

[6] Ma ZF Zhang Y Luo X Li X Li Y Liu S. Increased stressful impact among general population in mainland China amid the COVID-19 pandemic: a nationwide cross-sectional study conducted after Wuhan city's travel ban was lifted. Int J Soc Psychiatry. (2020) 66:770–9. 10.1177/0020764020935489 32564637 PMC7443962

[7] Rossi R Socci V Pacitti F Di Lorenzo G Di Marco A Siracusano A. Mental health outcomes among frontline and second-line health care workers during the coronavirus disease 2019 (COVID-19) pandemic in Italy. JAMA Netw Open. (2020) 3:e2010185. 10.1001/jamanetworkopen.2020.10185 32463467 PMC7256664

[8] Kang SJ Jung SI. Age-related morbidity and mortality among patients with COVID-19. Infect Chemother. (2020) 52:154–64. 10.3947/ic.2020.52.2.154 32537961 PMC7335648

[9] Zhou F Yu T Du R Fan G Liu Y Liu Z. Clinical course and risk factors for mortality of adult inpatients with COVID-19 in Wuhan, China: a retrospective cohort study. Lancet. (2020) 395:1054–62. 10.1016/S0140-6736(20)30566-3 32171076 PMC7270627

[10] Wu Z McGoogan JM. Characteristics of and important lessons from the coronavirus disease 2019 (COVID-19) outbreak in China: summary of a report of 72 314 cases from the Chinese center for disease control and prevention. JAMA. (2020) 323:1239–42. 10.1001/jama.2020.2648 32091533

[11] Wang K Zuo P Liu Y Zhang M Zhao X Xie S. Clinical and laboratory predictors of in-hospital mortality in patients with COVID-19: a cohort study in Wuhan, China. Clin Infect Dis. (2020) 71:2079–88. 10.1093/cid/ciaa538 32361723 PMC7197616

[12] Jawid A. Protecting older adults during social distancing. Science. (2020) 368:145. 10.1126/science.abb7885 32273460

[13] Santini ZI Jose PE York Cornwell E Koyanagi A Nielsen L Hinrichsen C. Social disconnectedness, perceived isolation, and symptoms of depression and anxiety among older Americans (NSHAP): a longitudinal mediation analysis. Lancet Public health. (2020) 5:e62–70. 10.1016/S2468-2667(19)30230-0 31910981

[14] Armitage R Nellums LB. COVID-19 and the consequences of isolating the elderly. Lancet Public health. (2020) 5:e256. 10.1016/S2468-2667(20)30061-X 32199471 PMC7104160

[15] Gerst-Emerson K Jayawardhana J. Loneliness as a public health issue: the impact of loneliness on health care utilization among older adults. Am J Public Health. (2015) 105:1013–9. 10.2105/AJPH.2014.302427 25790413 PMC4386514

[16] Yang Y Li W Zhang Q Zhang L Cheung T Xiang Y. Mental health services for older adults in China during the COVID-19 outbreak. Lancet Psychiatry. (2020) 7:e19. 10.1016/S2215-0366(20)30079-1 32085843 PMC7128970

[17] Huang Y Wang Y Wang H Liu Z Yu X Yan J. Prevalence of mental disorders in China: a cross-sectional epidemiological study. Lancet Psychiatry. (2019) 6:211–24. 10.1016/S2215-0366(18)30511-X 30792114

[18] Whiteford HA Degenhardt L Rehm J Baxter AJ Ferrari AJ Erskine HE. Global burden of disease attributable to mental and substance use disorders: findings from the global burden of disease study 2010. Lancet. (2013) 382:1575–86. 10.1016/S0140-6736(13)61611-6 23993280

[19] WHO. Guidance on Routine Immunization Services During COVID-19 Pandemic in the WHO European Region. (2020). Available online at: https://www.euro.who.int/en/health-topics/communicable-diseases/hepatitis/publications/2020/guidance-on-routine-immunization-services-during-covid-19-pandemic-in-the-who-european-region,-20-march-2020-produced-by-whoeurope. (accessed August 16, 2020).

[20] Lau AL Chi I Cummins RA Lee TM Chou KL Chung LW. The SARS (severe acute respiratory syndrome) pandemic in Hong Kong: effects on the subjective wellbeing of elderly and younger people. Aging Ment Health. (2008) 12:746–60. 10.1080/13607860802380607 19023726

[21] Chiu HF Lam LC Li SW Chiu E. SARS and psychogeriatrics: perspective and lessons from Hong Kong. Int J Geriatr Psychiatry. (2003) 18:871–3. 10.1002/gps.1003 14533118

[22] Meng H Xu Y Dai J Zhang Y Liu B Yang H. Analyze the psychological impact of COVID-19 among the elderly population in China and make corresponding suggestions. Psychiatry Res. (2020) 289:112983. 10.1016/j.psychres.2020.112983 32388175 PMC7151427

[23] Bruine de Bruin W. Age differences in COVID-19 risk perceptions and mental health: evidence from a national US survey conducted in March 2020. J Gerontol B Psychol Sci Soc Sci. (2020) 76:e24–9. 10.1093/geronb/gbaa074 PMC7542924 32470120

[24] Shi L Lu Z Que J Huang X Liu L Ran M. Prevalence of and risk factors associated with mental health symptoms among the general population in China during the coronavirus disease 2019 pandemic. JAMA Netw Open. (2020) 3:e2014053. 10.1001/jamanetworkopen.2020.14053 32609353 PMC7330717

[25] Sun X Li Y Yu C Li L. Reliability and validity of depression scales of Chinese version: a systematic review. Chin J Epidemiol. (2017) 38:110–6. 10.3760/cma.j.issn.0254-6450.2017.01.021 28100388

[26] He X Li C Qian J Cui H Wu W. Reliability and validity of a generalized anxiety disorder scale in general hospital outpatients. Shanghai Arch Psychiatry. (2010) 22:200–3. 10.3969/j.issn.1002-0829.2010.04.002

[27] Bai C Ji D Chen L Li L Wang C. Reliability and validity of insomnia severity index in clinical insomnia patients. Chin J Prac Nurs. (2018) 34:2182–6. 10.3760/cma.j.issn.1672-7088.2018.28.005

[28] Bryant RA Moulds ML Guthrie RM. Acute stress disorder scale: a self-report measure of acute stress disorder. Psychol Assess. (2000) 12:61–8. 10.1037/1040-3590.12.1.61 10752364

[29] Zhang Y Liang W Chen Z Zhang H Zhang J Weng X. Validity and reliability of patient health questionnaire-9 and patient health questionnaire-2 to screen for depression among college students in China. Asia Pac Psychiatry. (2013) 5:268–75. 10.1111/appy.12103 24123859

[30] Yu D. Insomnia severity index: psychometric properties with Chinese community-dwelling older people. J Adv Nurs. (2010) 66:2350–9. 10.1111/j.1365-2648.2010.05394.x 20722803

[31] Hu Y Chen Y Zheng Y You C Tan J Hu L. Factors related to mental health of inpatients with COVID-19 in Wuhan, China. Brain Behav Immun. (2020) 89:587–93. 10.1016/j.bbi.2020.07.016 32681866 PMC7362867

[32] Wang C Pan R Wan X Tan Y Xu L Ho CS. Immediate psychological responses and associated factors during the initial stage of the 2019 coronavirus disease (COVID-19) epidemic among the general population in China. Int J Environ Res Public Health. (2020) 17:1729. 10.3390/ijerph17051729 32155789 PMC7084952

[33] Li S Wang Y Xue J Zhao N Zhu T. The Impact of COVID-19 epidemic declaration on psychological consequences: a study on active Weibo users. Int J Environ Res Public Health. (2020) 17:2032. 10.3390/ijerph17062032 32204411 PMC7143846

[34] González-Sanguino C Ausín B Castellanos M Saiz J López-Gómez A Ugidos C. Mental health consequences during the initial stage of the 2020 coronavirus pandemic (COVID-19) in Spain. Brain Behav Immun. (2020) 87:172–6. 10.1016/j.bbi.2020.05.040 32405150 PMC7219372

[35] Stanton R To QG Khalesi S Williams SL Alley SJ Thwaite TL. Depression, anxiety and stress during covid-19: associations with changes in physical activity, sleep, tobacco and alcohol use in Australian adults. Int J Environ Res Public Health. (2020) 17:4065. 10.3390/ijerph17114065 32517294 PMC7312903

[36] Faye C McGowan JC Denny CA David DJ. Neurobiological mechanisms of stress resilience and implications for the aged population. Curr Neuropharmacol. (2018) 16:234–70. 10.2174/1570159X15666170818095105 28820053 PMC5843978

[37] Chen LK. Older adults and COVID-19 pandemic: resilience matters. Arch Gerontol Geriatr. (2020) 89:104124. 10.1016/j.archger.2020.104124 32474351 PMC7247489

[38] Brooks SK Webster RK Smith LE Woodland L Wessely S Greenberg N. The psychological impact of quarantine and how to reduce it: rapid review of the evidence. Lancet. (2020) 395:912–20. 10.1016/S0140-6736(20)30460-8 32112714 PMC7158942

[39] Liu J Bao Y Huang X Shi J Lu L. Mental health considerations for children quarantined because of COVID-19. Lancet Child Adolesc Health. (2020) 43:327–31. 10.1016/S2352-4642(20)30096-1 32224303 PMC7118598

[40] Goethals L Barth N Guyot J Hupin D Celarier T Bongue B. Impact of home quarantine on physical activity among older adults living at home during the COVID-19 pandemic: qualitative interview study. JMIR Aging. (2020) 3:e19007. 10.2196/19007 32356777 PMC7207013

[41] Cole SW Capitanio JP Chun K Arevalo JM Ma J Cacioppo JT. Myeloid differentiation architecture of leukocyte transcriptome dynamics in perceived social isolation. Proc Natl Acad Sci USA. (2015) 112:15142–7. 10.1073/pnas.1514249112 26598672 PMC4679065

[42] Banskota S Healy M Goldberg EM. 15 Smartphone apps for older adults to use while in isolation during the covid-19 pandemic. West J Emerg Med. (2020) 21:514–25. 10.5811/westjem.2020.4.47372 32302279 PMC7234684

[43] Behan C. The benefits of meditation and mindfulness practices during times of crisis such as COVID-19. Ir J Psychol Med. (2020) 37:256–8. 10.1017/ipm.2020.38 32406348 PMC7287297

[44] Dkhar SA Quansar R Saleem SM Khan SMS. Knowledge, attitude, and practices related to COVID-19 pandemic among social media users in J&K, India. Indian J Public Health. (2020) 64:S205–10. 10.4103/ijph.IJPH_469_20 32496256

[45] Bukhsh A Hussain S Rehman IU Mallhi TH Khan YH Khaliel AM. Awareness and perception of seasonal influenza (Flu) among health science and non-health science university students in Pakistan: a nationwide survey. Pak J Pharm Sci. (2019) 32:1789–96. 31680074

[46] Chan EY Cheng CK Tam G Huang Z Lee P. Knowledge, attitudes, and practices of Hong Kong population towards human A/H7N9 influenza pandemic preparedness, China, 2014. BMC Public Health. (2015) 15:943. 10.1186/s12889-015-2245-9 26395243 PMC4579795

[47] Que J Shi L Deng J Liu J Zhang L Wu S. Psychological impact of the covid-19 pandemic on healthcare workers: a cross-sectional study in China. Gen Psychiatr. (2020) 33:e100259. 10.1136/gpsych-2020-100259 32596640 PMC7299004

[48] Pan Y Li X Yang G Fan J Tang Y Zhao J. Serological immunochromatographic approach in diagnosis with SARS-CoV-2 infected COVID-19 patients. J Infect. (2020) 81:e28–32. 10.1016/j.jinf.2020.03.051 32283141 PMC7195339

[49] Ribeiro WS Bauer A Andrade MCR York-Smith M Pan PM Pingani L. Income inequality and mental illness-related morbidity and resilience: a systematic review and meta-analysis. Lancet Psychiatry. (2017) 4:554–62. 10.1016/S2215-0366(17)30159-1 28552501

[50] Lund C De Silva M Plagerson S Cooper S Chisholm D Das J. Poverty and mental disorders: breaking the cycle in low-income and middle-income countries. Lancet. (2011) 378:1502–14. 10.1016/S0140-6736(11)60754-X 22008425

[51] Li Y Mutchler JE. Older adults and the economic impact of the COVID-19 pandemic. J Aging Soc Policy. (2020) 32:477–87. 10.1080/08959420.2020.1773191 32543304

[52] Paul KI Moser K. Unemployment impairs mental health: meta-analyses. J Vocat Behav. (2009) 74:264–82. 10.1016/j.jvb.2009.01.001

[53] Picaza Gorrochategi M Eiguren Munitis A Dosil Santamaria M Ozamiz Etxebarria N. Stress, anxiety, and depression in people aged over 60 in the covid-19 outbreak in a sample collected in northern Spain. Am J Geriatr Psychiatry. (2020) 28:993–8. 10.1016/j.jagp.2020.05.022 32576424 PMC7261426

