# Supplementary material for: Mental Health Status of Late-Middle-Aged Adults in China During the Coronavirus Disease 2019 Pandemic
Source: Front Public Health. 2021 May 26;9:643988. doi: 10.3389/fpubh.2021.643988 (PMC8187778; doi:10.3389/fpubh.2021.643988)
Supplement: Supplementary file 1 [file Table_1.DOCX]

**Supplementary Table 1. Categories of severity of anxiety, depression, insomnia, and acute stress in quarantined late-middle-age adults (n=737) stratified by pandemic-related factors.**

|  | **Depression**^a^ | | | | **Anxiety**^b^ | | | | **Insomnia**^c^ | | | | **Acute stress**^d^ | | |
| --- | --- | --- | --- | --- | --- | --- | --- | --- | --- | --- | --- | --- | --- | --- | --- |
|  | **Participants, No. (%)** | | | | **Participants, No. (%)** | | | | **Participants, No. (%)** | | | | **Participants, No. (%)** | | |
| **Variables** | **Normal** | **Mild** | **Moderate to severe** | *p*^e^ | **Normal** | **Mild** | **Moderate to severe** | *p*^e^ | **Normal** | **Mild** | **Moderate to severe** | *p*^e^ | **Normal** | **Stressed** | *p*^e^ |
| **Overall** | 545 (73.9) | 108 (14.7) | 84 (11.4) |  | 492 (66.8) | 148 (20.1) | 97 (13.2) |  | 498 (67.6) | 181 (24.6) | 58 (7.9) |  | 546 (74.1) | 191 (25.9) |  |
| **Gender** |  |  |  | 0.905 |  |  |  | 0.829 |  |  |  | 0.169 |  |  | 0.964 |
| Male | 244 (74.2) | 49 (14.9) | 36 (10.9) |  | 221 (67.2) | 68 (20.7) | 40 (12.2) |  | 231 (70.2) | 80 (24.3) | 18 (5.5) |  | 244 (74.2) | 85 (25.8) |  |
| Female | 301 (73.8) | 59 (14.5) | 48 (11.8) |  | 271 (66.4) | 80 (19.6) | 57 (14.0) |  | 267 (65.4) | 101 (24.8) | 40 (9.8) |  | 302 (74.0) | 106 (26.0) |  |
| **Living area** |  |  |  | 0.952 |  |  |  | 0.477 |  |  |  | 0.352 |  |  | 0.678 |
| Urban | 516 (73.9) | 104 (14.9) | 78 (11.2) |  | 468 (67.0) | 138 (19.8) | 92 (13.2) |  | 469 (67.2) | 173 (24.8) | 56 (8.0) |  | 516 (73.9) | 182 (26.1) |  |
| Rural | 29 (74.4) | 4 (10.3) | 6 (15.4) |  | 24 (61.5) | 10 (25.6) | 5 (12.8) |  | 29 (74.4) | 8 (20.5) | 2 (5.1) |  | 30 (76.9) | 9 (23.1) |  |
| **Level of education** |  |  |  | 0.148 |  |  |  | 0.714 |  |  |  | 0.783 |  |  | 0.118 |
| Less than college | 199 (77.1) | 35 (13.6) | 24 (9.3) |  | 170 (65.9) | 56 (21.7) | 32 (12.4) |  | 176 (68.2) | 68 (26.4) | 14 (5.4) |  | 200 (77.5) | 58 (22.5) |  |
| College degree or higher | 346 (72.2) | 73 (15.2) | 60 (12.5) |  | 322 (67.2) | 92 (19.2) | 65 (13.6) |  | 322 (67.2) | 113 (23.6) | 44 (9.2) |  | 346 (72.2) | 133 (27.8) |  |
| **Marital status** |  |  |  | 0.556 |  |  |  | 0.341 |  |  |  | 0.813 |  |  | 0.526 |
| Married | 504 (74.2) | 98 (14.4) | 77 (11.3) |  | 450 (66.3) | 139 (20.5) | 90 (13.3) |  | 458 (67.5) | 166 (24.4) | 55 (8.1) |  | 501 (73.8) | 178 (26.2) |  |
| Unmarried | 41 (70.7) | 10 (17.2) | 7 (12.1) |  | 42 (72.4) | 9 (15.5) | 7 (12.1) |  | 40 (69.0) | 15 (25.9) | 3 (5.2) |  | 45 (77.6) | 13 (22.4) |  |
| **Monthly family income, ¥**^f^ |  |  |  | 0.014 |  |  |  | 0.015 |  |  |  | 0.284 |  |  | 0.091 |
| 0-4999 | 123 (69.1) | 31 (17.4) | 24 (13.5) |  | 108 (60.7) | 40 (22.5) | 30 (16.9) |  | 115 (64.6) | 48 (27.0) | 15 (8.4) |  | 128 (71.9) | 50 (28.1) |  |
| 5000-11999 | 260 (72.2) | 57 (15.8) | 43 (11.9) |  | 236 (65.6) | 76 (21.1) | 48 (13.3) |  | 240 (66.7) | 91 (25.3) | 29 (8.1) |  | 259 (71.9) | 101 (28.1) |  |
| ≥ 12000 | 162 (81.4) | 20 (10.1) | 17 (8.5) |  | 148 (74.4) | 32 (16.1) | 19 (9.5) |  | 143 (71.9) | 42 (21.1) | 14 (7.0) |  | 159 (79.9) | 40 (20.1) |  |
| **History of chronic disease** |  |  |  | 0.063 |  |  |  | 0.210 |  |  |  | 0.026 |  |  | 0.060 |
| Yes | 114 (74.5) | 22 (14.4) | 17 (11.1) |  | 102 (66.7) | 30 (19.6) | 21 (13.7) |  | 97 (63.4) | 40 (26.1) | 16 (10.5) |  | 114 (74.5) | 39 (25.5) |  |
| No | 415 (74.8) | 76 (13.7) | 64 (11.5) |  | 375 (67.6) | 108 (19.5) | 72 (13.0) |  | 387 (69.7) | 129 (23.2) | 39 (7.0) |  | 416 (75.0) | 139 (25.0) |  |
| Unknown | 16 (55.2) | 10 (34.5) | 3 (10.3) |  | 15 (51.7) | 10 (34.5) | 4 (13.8) |  | 14 (48.3) | 12 (41.4) | 3 (10.3) |  | 16 (55.2) | 13 (44.8) |  |
| **History of psychiatric disorders** |  |  |  | 0.111 |  |  |  | 0.101 |  |  |  | 0.198 |  |  | 0.248 |
| Yes | 1 (33.3) | 0 | 2 (66.7) |  | 1 (33.3) | 0 | 2 (66.7) |  | 2 (66.7) | 0 | 1 (33.3) |  | 1 (33.3) | 2 (66.7) |  |
| No | ‘541 (74.3) | 107 (14.7) | 80 (11.0) |  | 489 (67.2) | 148 (20.3) | 91 (12.5) |  | 494 (67.9) | 179 (24.6) | 55 (7.6) |  | 541 (74.3) | 187 (25.7) |  |
| Unknown | 3 (50.0) | 1 (16.7) | 2 (33.3) |  | 2 (33.3) | 0 | 4 (66.7) |  | 2 (33.3) | 2 (33.3) | 2 (33.3) |  | 4 (66.7) | 2 (33.3) |  |
| **Family history of psychiatric disorders** |  |  |  | 0.034 |  |  |  | 0.069 |  |  |  | 0.759 |  |  | 0.675 |
| Yes | 0 | 2 (100) | 0 |  | 0 | 1 (50.0) | 1 (50.0) |  | 1 (50.0) | 1 (50.0) | 0 |  | 1 (50.0) | 1 (50.0) |  |
| No | 539 (74.3) | 105 (14.5) | 81 (11.2) |  | 487 (67.2) | 146 (20.1) | 92 (12.7) |  | 491 (67.7) | 178 (24.6) | 56 (7.7) |  | 537 (74.1) | 188 (25.9) |  |
| Unknown | 6 (60.0) | 1 (10.0) | 3 (30.0) |  | 5 (50.0) | 1 (10.0) | 4 (40.0) |  | 6 (60.0) | 2 (20.0) | 2 (20.0) |  | 8 (80.0) | 2 (20.0) |  |
| **Are you a frontline worker?** |  |  |  | 0.705 |  |  |  | 0.407 |  |  |  | 0.418 |  |  | 0.953 |
| Yes | 79 (72.5) | 11 (10.1) | 19 (17.4) |  | 69 (63.3) | 20 (18.3) | 20 (18.3) |  | 70 (64.2) | 29 (26.6) | 10 (9.2) |  | 81 (74.3) | 28 (25.7) |  |
| No | 466 (74.2) | 97 (15.4) | 65 (10.4) |  | 423 (67.4) | 128 (20.4) | 77 (12.3) |  | 428 (68.2) | 152 (24.2) | 48 (7.6) |  | 465 (74.0) | 163 (26.6) |  |
| **Have any of your family members or friends been infected with COVID-19?** |  |  |  | 0.060 |  |  |  | 0.177 |  |  |  | 0.160 |  |  | 0.006 |
| Yes | 3 (42.9) | 0 | 4 (57.1) |  | 3 (42.9) | 0 | 4 (57.1) |  | 3 (42.9) | 1 (14.3) | 3 (42.9) |  | 2 (28.6) | 5 (71.4) |  |
| No | 542 (74.2) | 108 (14.8) | 80 (11.0) |  | 489 (67.0) | 148 (20.3) | 93 (12.7) |  | 495 (67.8) | 180 (24.7) | 55 (7.5) |  | 544 (74.5) | 186 (25.5) |  |
| **Are any of your family members or friends frontline workers?** |  |  |  | 0.995 |  |  |  | 0.883 |  |  |  | 0.247 |  |  | 0.955 |
| Yes | 196 (74.0) | 37 (14.0) | 32 (12.1) |  | 176 (66.4) | 48 (18.1) | 41 (15.5) |  | 172 (64.9) | 70 (26.4) | 23 (8.7) |  | 196 (74.0) | 69 (26.0) |  |
| No | 349 (73.9) | 71 (15.0) | 52 (11.0) |  | 316 (66.9) | 100 (21.2) | 56 (11.9) |  | 326 (69.1) | 111 (23.5) | 35 (7.4) |  | 350 (74.2) | 122 (25.8) |  |
| **Are you in Hubei Province now?** |  |  |  | 0.662 |  |  |  | 0.352 |  |  |  | 0.725 |  |  | 0.462 |
| Yes | 59 (72.0) | 13 (15.9) | 10 (12.2) |  | 51 (62.2) | 21 (25.6) | 10 (12.2) |  | 54 (65.9) | 21 (25.6) | 7 (8.5) |  | 58 (70.7) | 24 (29.3) |  |
| No | 486 (74.2) | 95 (14.5) | 74 (11.3) |  | 441 (67.3) | 127 (19.4) | 87 (13.3) |  | 444 (67.8) | 160 (24.4) | 51 (7.8) |  | 488 (74.5) | 167 (25.5) |  |
| **Are you back to work now?** |  |  |  | 0.714 |  |  |  | 0.553 |  |  |  | 0.378 |  |  | 0.090 |
| Absent from work | 141 (73.4) | 29 (15.1) | 22 (11.5) |  | 129 (67.2) | 36 (18.8) | 27 (14.1) |  | 121 (63.0) | 52 (27.1) | 19 (9.9) |  | 138 (71.9) | 54 (28.1) |  |
| Always at work | 90 (77.6) | 16 (13.8) | 10 (8.6) |  | 77 (66.4) | 24 (20.7) | 15 (12.9) |  | 78 (67.2) | 27 (23.3) | 11 (9.5) |  | 81 (69.8) | 35 (30.2) |  |
| Not back to work | 142 (71.7) | 33 (16.7) | 23 (11.6) |  | 125 (63.1) | 46 (23.2) | 27 (13.6) |  | 135 (68.2) | 48 (24.2) | 15 (7.6) |  | 142 (71.7) | 56 (28.3) |  |
| Back to work | 172 (74.5) | 30 (13.0) | 29 (12.6) |  | 161 (69.7) | 42 (18.2) | 28 (12.1) |  | 164 (71.0) | 54 (23.4) | 13 (5.6) |  | 185 (80.1) | 46 (19.9) |  |
| **Are you likely to be exposed to other people at work?** |  |  |  | 0.642 |  |  |  | 0.847 |  |  |  | 0.587 |  |  | 0.337 |
| Exposed to patients infected with COVID-19 | 18 (72.0) | 1 (4.0) | 6 (24.0) |  | 18 (72.0) | 2 (8.0) | 5 (20.0) |  | 15 (60.0) | 8 (32.0) | 2 (8.0) |  | 19 (76.0) | 6 (24.0) |  |
| Exposed to patients with other diseases | 8 (72.7) | 1 (9.1) | 2 (18.2) |  | 8 (72.7) | 2 (18.2) | 1 (9.1) |  | 8 (72.7) | 3 (27.3) | 0 |  | 9 (81.8) | 2 (18.2) |  |
| Exposed to general people | 141 (78.8) | 20 (11.2) | 18 (10.1) |  | 125 (69.8) | 30 (16.8) | 24 (13.4) |  | 128 (71.5) | 42 (23.5) | 9 (5.0) |  | 142 (79.3) | 37 (20.7) |  |
| Not at work, work at home, or without exposure to people at work | 350 (74.2) | 79 (16.7) | 43 (9.1) |  | 316 (66.9) | 104 (22.0) | 52 (11.0) |  | 318 (67.4) | 113 (23.9) | 41 (8.7) |  | 343 (72.7) | 129 (27.3) |  |
| **Do you live in a community that restricts people’s access?** |  |  |  | 0.644 |  |  |  | 0.399 |  |  |  | 0.752 |  |  | 0.371 |
| Yes | 524 (73.8) | 106 (14.9) | 80 (11.3) |  | 476 (67.0) | 142 (20.0) | 92 (13.0) |  | 479 (67.5) | 173 (24.4) | 58 (8.2) |  | 524 (73.8) | 186 (26.2) |  |
| No | 21 (77.8) | 2 (7.4) | 4 (14.8) |  | 16 (59.3) | 6 (22.2) | 5 (18.5) |  | 19 (70.4) | 8 (29.6) | 0 |  | 22 (81.5) | 5 (18.5) |  |
| **Were there any traffic restrictions** **in your area during the pandemic?** |  |  |  | 0.243 |  |  |  | 0.445 |  |  |  | 0.648 |  |  | 0.623 |
| Yes | 433 (73.0) | 91 (15.3) | 69 (11.6) |  | 392 (66.1) | 121 (20.4) | 80 (13.5) |  | 403 (68.0) | 147 (24.8) | 43 (7.3) |  | 437 (73.7) | 156 (26.3) |  |
| No | 112 (77.8) | 17 (11.8) | 15 (10.4) |  | 100 (69.4) | 27 (18.8) | 17 (11.8) |  | 95 (66.0) | 34 (23.6) | 15 (10.4) |  | 109 (75.7) | 35 (24.3) |  |
| **Are you highly concerned about the COVID-19 pandemic?** |  |  |  | 0.015 |  |  |  | 0.231 |  |  |  | 0.765 |  |  | 0.978 |
| Yes | 518 (75.0) | 99 (14.3) | 74 (10.7) |  | 465 (67.3) | 137 (19.8) | 89 (12.9) |  | 466 (67.4) | 169 (23.5) | 56 (8.1) |  | 512 (74.1) | 179 (25.9) |  |
| No | 27 (58.7) | 9 (19.6) | 10 (21.7) |  | 27 (58.7) | 11 (23.9) | 8 (17.4) |  | 32 (69.6) | 12 (26.1) | 2 (4.3) |  | 34 (73.9) | 12 (26.1) |  |
| **Do you have a good understanding of the COVID-19 pandemic?** |  |  |  | 0.004 |  |  |  | 0.017 |  |  |  | 0.397 |  |  | 0.296 |
| Yes | 493 (75.6) | 95 (14.6) | 64 (9.8) |  | 445 (68.3) | 129 (19.8) | 78 (12.0) |  | 444 (68.1) | 160 (24.5) | 48 (7.4) |  | 487 (74.7) | 165 (25.3) |  |
| No | 52 (61.2) | 13 (15.3) | 20 (23.5) |  | 47 (55.3) | 19 (22.4) | 19 (22.4) |  | 54 (63.5) | 21 (24.7) | 10 (11.8) |  | 59 (69.4) | 26 (30.6) |  |

COVID-19, coronavirus disease 2019. ^a^ Scores of 5-9 on the Patient Health Questionnaire–9 were defined as mild depression, and scores of ≥ 10 were defined as moderate-to-severe depression. ^b^ Scores of 5-9 on the Generalized Anxiety Disorder–7 were defined as mild anxiety, and scores of ≥ 10 were defined as moderate-to-severe anxiety. ^c^ Scores of 8-14 on the Insomnia Severity Index were defined as subthreshold insomnia, and scores of ≥ 15 were defined as moderate-to-severe insomnia. ^d^ Acute stress symptoms were defined as having an Acute Stress Disorder Scale dissociative cluster score of ≥ 9 and cumulative re-experiencing, avoidance, and arousal cluster scores of ≥ 28. ^e^ *χ^2^* tests were used to compare the prevalence of mild-to-severe mental health symptoms in different populations. ^f^ 1 ¥ = USD$0.14.

**Supplementary Table 2. Multivariable regression analysis of risk factors associated with symptoms of depression, anxiety, insomnia, and acute stress in quarantined late-middle-age adults (n=687) during the COVID-19 pandemic.**

|  | **Depression** | | **Anxiety** | | **Insomnia** | | **Acute stress** | |
| --- | --- | --- | --- | --- | --- | --- | --- | --- |
| **Variable** | **AOR (95% CI)** | ***p*** | **AOR (95% CI)** | ***p*** | **AOR (95% CI)** | ***p*** | **AOR (95% CI)** | ***p*** |
| **Gender** |  |  |  |  |  |  |  |  |
| Male | 0.97 (0.66-1.43) | 0.879 | 0.90 (0.63-1.28) | 0.546 | 0.82 (0.57-1.17) | 0.274 | 1.07 (0.73-1.57) | 0.742 |
| Female | 1 [Reference] |  | 1 [Reference] |  | 1 [Reference] |  | 1 [Reference] |  |
| **Living area** |  |  |  |  |  |  |  |  |
| Urban | 0.91 (0.41-2.06) | 0.826 | 0.79 (0.38-1.64) | 0.526 | 1.26 (0.57-2.81) | 0.566 | 1.00 (0.44-2.28) | 0.995 |
| Rural | 1 [Reference] |  | 1 [Reference] |  | 1 [Reference] |  | 1 [Reference] |  |
| **Level of education** |  |  |  |  |  |  |  |  |
| Less than college | 0.53 (0.34-0.83) | 0.005 | 0.90 (0.61-1.33) | 0.595 | 0.92 (0.62-1.37) | 0.685 | 0.64 (0.41-0.98) | 0.040 |
| College degree or higher | 1 [Reference] |  | 1 [Reference] |  | 1 [Reference] |  | 1 [Reference] |  |
| **Marital status** |  |  |  |  |  |  |  |  |
| Married | 0.88 (0.46-1.72) | 0.716 | 1.61 (0.83-3.14) | 0.161 | 1.21 (0.64-2.29) | 0.558 | 1.36 (0.67-2.73) | 0.396 |
| Unmarried | 1 [Reference] |  | 1 [Reference] |  | 1 [Reference] |  | 1 [Reference] |  |
| **Monthly family income, ¥**^a^ |  |  |  |  |  |  |  |  |
| 0-4999 | 2.77 (1.55-4.95) | 0.001 | 2.27 (1.35-3.84) | 0.002 | 1.63 (0.97-2.75) | 0.066 | 1.95 (1.10-3.44) | 0.022 |
| 5000-11999 | 1.92 (1.19-3.08) | 0.008 | 1.68 (1.10-2.56) | 0.017 | 1.49 (0.98-2.26) | 0.060 | 1.74 (1.10-2.75) | 0.017 |
| ≥ 12000 | 1 [Reference] |  | 1 [Reference] |  | 1 [Reference] |  | 1 [Reference] |  |
| **History of chronic disease** |  |  |  |  |  |  |  |  |
| Yes | 1.01 (0.64-1.61) | 0.960 | 1.02 (0.67-1.55) | 0.917 | 1.42 (0.95-2.13) | 0.091 | 0.96 (0.61-1.51) | 0.866 |
| No | 1 [Reference] |  | 1 [Reference] |  | 1 [Reference] |  | 1 [Reference] |  |
| Unknown | 2.60 (1.10-6.14) | 0.029 | 1.81 (0.79-4.15) | 0.164 | 2.16 (0.93-4.99) | 0.072 | 2.29 (0.99-5.31) | 0.054 |
| **History of psychiatric disorders** |  |  |  |  |  |  |  |  |
| Yes | 2.56 (0.41-47.31) | 0.527 | 1.74 (0.10-31.47) | 0.706 | 1.50 (0.08-26.67) | 0.783 | 2.78 (0.15-50.93) | 0.492 |
| No | 1 [Reference] |  | 1 [Reference] |  | 1 [Reference] |  | 1 [Reference] |  |
| Unknown | 1.15 (0.09-14.42) | 0.916 | 2.81 (0.25-31.27) | 0.401 | 17.13 (0.80-367.52) | 0.069 | 3.93 (0.26-59.72) | 0.325 |
| **Family history of psychiatric disorders** |  |  |  |  |  |  |  |  |
| Yes | NA | NA | NA | NA | 2.59 (0.15-45.81) | 0.516 | 2.87 (0.16-51.78) | 0.492 |
| No | 1 [Reference] |  | 1 [Reference] |  | 1 [Reference] |  | 1 [Reference] |  |
| Unknown | 1.49 (0.23-9.70) | 0.679 | 1.14 (0.19-6.83) | 0.885 | 0.57 (0.06-5.20) | 0.622 | 0.43 (0.04-4.62) | 0.484 |
| **Are you a frontline worker?** |  |  |  |  |  |  |  |  |
| Yes | 1.11 (0.60-2.03) | 0.743 | 1.26 (0.72-2.19) | 0.421 | 1.20 (0.69-2.10) | 0.511 | 1.05 (0.58-1.92) | 0.867 |
| No | 1 [Reference] |  | 1 [Reference] |  | 1 [Reference] |  | 1 [Reference] |  |
| **Have any of your family members or friends been infected with COVID-19?** |  |  |  |  |  |  |  |  |
| Yes | 4.09 (0.84-19.97) | 0.082 | 2.79 (0.57-13.52) | 0.204 | 2.32 (0.48-11.16) | 0.296 | 7.27(1.29-40.95) | 0.024 |
| No | 1 [Reference] |  | 1 [Reference] |  | 1 [Reference] |  | 1 [Reference] |  |
| **Are any of your family members or friends frontline workers?** |  |  |  |  |  |  |  |  |
| Yes | 1.10 (0.73-1.67) | 0.640 | 1.05 (0.72-1.53) | 0.808 | 1.30 (0.90-1.90) | 0.167 | 1.11 (0.74-1.67) | 0.604 |
| No | 1 [Reference] |  | 1 [Reference] |  | 1 [Reference] |  | 1 [Reference] |  |
| **Are you in Hubei Province now?** |  |  |  |  |  |  |  |  |
| Yes | 1.00 (0.55-1.85) | 0.990 | 0.94 (0.53-1.65) | 0.827 | 0.86 (0.48-1.52) | 0.601 | 0.88 (0.48-1.60) | 0.673 |
| No | 1 [Reference] |  | 1 [Reference] |  | 1 [Reference] |  | 1 [Reference] |  |
| **Are you back to work now?** |  |  |  |  |  |  |  |  |
| Absent from work | 0.76 (0.40-1.42) | 0.388 | 0.82 (0.46-1.47) | 0.501 | 1.37 (0.77-2.46) | 0.290 | 1.50 (0.80-2.82) | 0.208 |
| Always at work | 0.68 (0.37-1.23) | 0.201 | 1.02 (0.60-1.74) | 0.932 | 1.04 (0.61-1.77) | 0.893 | 1.53 (0.86-2.70) | 0.146 |
| Not back to work | 0.85 (0.46-1.56) | 0.589 | 1.01 (0.57-1.79) | 0.969 | 1.03 (0.58-1.85) | 0.914 | 1.34 (0.72-2.48) | 0.356 |
| Back to work | 1 [Reference] |  | 1 [Reference] |  | 1 [Reference] |  | 1 [Reference] |  |
| **Are you likely to be exposed to other people at work?** |  |  |  |  |  |  |  |  |
| Exposed to patients infected with COVID-19 | 0.94 (0.33-2.68) | 0.903 | 0.60 (0.22-1.66) | 0.326 | 1.42 (0.55-3.66) | 0.470 | 0.77 (0.25-2.32) | 0.639 |
| Exposed to patients with other diseases | 1.22 (0.28-5.27) | 0.792 | 0.76 (0.18-3.25) | 0.710 | 0.95 (0.23-4.01) | 0.946 | 0.71 (0.14-3.73) | 0.686 |
| Exposed to general people | 0.79 (0.44-1.40) | 0.414 | 0.87 (0.51-1.48) | 0.610 | 0.96 (0.56-1.65) | 0.878 | 0.81 (0.45-1.45) | 0.473 |
| Not at work, work at home, or without exposure to people at work | 1 [Reference] |  | 1 [Reference] |  | 1 [Reference] |  | 1 [Reference] |  |
| **Do you live in a community that restricts people’s access?** |  |  |  |  |  |  |  |  |
| Yes | 1.91 (0.51-7.09) | 0.335 | 0.90 (0.34-2.41) | 0.840 | 1.63 (0.58-4.60) | 0.353 | 1.57 (0.49-5.03) | 0.446 |
| No | 1 [Reference] |  | 1 [Reference] |  | 1 [Reference] |  | 1 [Reference] |  |
| **Were there any traffic restrictions in your area during the pandemic?** |  |  |  |  |  |  |  |  |
| Yes | 1.19 (0.72-1.98) | 0.504 | 1.10 (0.70-1.74) | 0.683 | 0.80 (0.52-1.25) | 0.336 | 0.99 (0.61-1.61) | 0.978 |
| No | 1 [Reference] |  | 1 [Reference] |  | 1 [Reference] |  | 1 [Reference] |  |
| **Are you highly concerned about the COVID-19 pandemic?** |  |  |  |  |  |  |  |  |
| Yes | 0.56 (0.26-1.21) | 0.139 | 0.83 (0.39-1.74) | 0.619 | 1.30 (0.59-2.87) | 0.520 | 0.97 (0.43-5.03) | 0.948 |
| No | 1 [Reference] |  | 1 [Reference] |  | 1 [Reference] |  | 1 [Reference] |  |
| **Do you have a good understanding of the COVID-19 pandemic?** |  |  |  |  |  |  |  |  |
| Yes | 0.86 (0.47-1.59) | 0.639 | 0.87 (0.49-1.55) | 0.640 | 1.03 (0.57-1.87) | 0.923 | 1.06 (0.57-2.00) | 0.849 |
| No | 1 [Reference] |  | 1 [Reference] |  | 1 [Reference] |  | 1 [Reference] |  |

AOR, adjusted odds ratio; COVID-19, coronavirus disease 2019. ^a^ 1 ¥ = USD$0.14.
